# Early life stress and development: potential mechanisms for adverse outcomes

**DOI:** 10.1186/s11689-020-09337-y

**Published:** 2020-12-16

**Authors:** Karen E. Smith, Seth D. Pollak

**Affiliations:** Department of Psychology and Waisman Center, University of Wisconsin–Madison, 1500 S Highland Blvd, Rm 399, Madison, WI 53705 USA

**Keywords:** Early life stress, Early adversity, Neurobiological development, Developmental disorders

## Abstract

**Background:**

Chronic and/or extreme stress in early life, often referred to as early adversity, childhood trauma, or early life stress, has been associated with a wide range of adverse effects on development. However, while early life stress has been linked to negative effects on a number of neural systems, the specific mechanisms through which early life stress influences development and individual differences in children’s outcomes are still not well understood.

**Main text:**

The current paper reviews the existing literature on the neurobiological effects of early life stress and their ties to children’s psychological and behavioral development.

**Conclusions:**

Early life stress has persistent and pervasive effects on prefrontal–hypothalamic–amygdala and dopaminergic circuits that are at least partially mediated by alterations in hypothalamic–pituitary–adrenal axis function. However, to date, this research has primarily utilized methods of assessment that focus solely on children’s event exposures. Incorporating assessment of factors that influence children’s interpretation of stressors, along with stressful events, has the potential to provide further insight into the mechanisms contributing to individual differences in neurodevelopmental effects of early life stress. This can aid in further elucidating specific mechanisms through which these neurobiological changes influence development and contribute to risk for psychopathology and health disorders.

## Background

Early life experiences represent an important influence on children’s neural, behavioral, and psychological development, having long-lasting effects across a wide range of domains [1, 2]. Experience shapes neural plasticity and through this behavior and psychological processes throughout the lifespan [3, 4]. Infancy and early childhood are periods of particularly high rates of synaptic regrowth and remodeling in the brain, during which experience can have long-lasting effects on development [5, 6]. Neuroscience has greatly illuminated our understanding of how both positive and negative early life experiences affect brain development, with implications for children’s mental and physical health. In this paper, we review the literature examining the neurobiological effects of early experiences and discuss where there is a need for further research related to individual differences in children’s responses to their early environments.

An early experience that has garnered much attention is that of chronic and/or extreme stress in early life. Experiences of chronic and/or severe stress during early childhood, often also conceptualized as early life stress, childhood adversity, child maltreatment, or childhood trauma, have persistent and pervasive consequences for development [7, 8]. The term stress refers to the psychological response elicited when an individual perceives themselves to be under threat or challenge and is generally beneficial, eliciting a range of behavioral and physiological changes aimed at addressing the perceived threat. However, chronic and/or extreme stress results in extended activation of these psychological, behavioral, and physiological stress response systems leading to dysregulation and negative psychological and behavioral outcomes [9, 10]. Here, we use the term early life stress broadly to refer to stress occurring in childhood (prior to the age of 18). It is a term which encompasses many different kinds of adverse experiences a child might encounter, including, but not limited to, exposure to toxins, nutritional restriction, abuse, neglect, and limited family resources. Severe and chronic exposure to these types of situations has long-term negative consequences on a wide range of cognitive, emotional, and behavioral processes [11–13]. However, the neural mechanisms supporting these effects are less well understood, and advances in neuroscience are critical for uncovering causal mechanisms linking exposure to early life adverse experiences with well-being across the lifespan.

Below, we review the current state of the literature on the effects of early experiences of stress on neurobiological circuits and the implications these effects have for children’s development. We start by introducing two prevalent approaches toward conceptualizing early life stress and its effects on development. We then highlight common findings across these different approaches related to the neural effects of early life stress, with a particular focus on the effects on prefrontal–hippocampal–amygdala and dopaminergic circuits. Finally, we address opportunities for new ways in which to advance our understanding of the mechanisms through which early life stress shapes the developing brain, and in turn children’s health outcomes. Together, these data can inform the development of more effective and targeted interventions for at risk children.

## Main text

### Models for conceptualizing early life stress: elucidating neurobiological mechanisms

Researchers have employed a variety of models aimed at conceptualizing early life stress, with the goal of better elucidating the neurobiological mechanisms through which stress exerts effects on development. While the question of how to best conceptualize early childhood adversity and stress has shifted over time [14, 15], the two predominant models of early life stress fall into the categories of (1) General or “lumping” models, in which various types of stressors are treated as a heterogeneous, broad category, often labeled “adversity,” “early life stress,” or “negative life events” [16–19]; and 2) Specific or “splitting” models, which are based on the premise that different types of adversity each confer specific effects, and links to neurobiological or cognitive systems may be masked by heterogeneous samples [20–22]. Both types of models have provided a wealth of knowledge surrounding early childhood adversity and its effects on development and provided initial insight into some of the potential neurobiological mechanisms underlying these effects. However, there is still much the field does not understand about what bio-behavioral mechanisms account for individual patterns of developmental change following extreme adversity. In the following sections, we will review the literature supporting general and specific effects of early life stress on neurobiological systems.

#### Insights from general models

One common general approach to conceptualizing early adversity is that of cumulative measures of adversity. In this approach, individuals are queried about whether they experienced a pre-defined set of potential adverse events in childhood, and their total exposure to events from that list is summed [23, 24]. Examples of these methods include variations on Life Stressors Checklists [25] and the Adverse Child Experiences Scale (ACES) [18, 26]. This approach is based in animal literature that suggests repeated exposure to stress, regardless of type, through chronic activation of stress response systems (i.e., HPA, immune, and autonomic nervous system), alters neural synaptic plasticity leading to cognitive deficits, anxiety, and depressive-like behaviors, and poorer health [9, 27]. Similarly, in humans, cumulative measures of adversity have been linked to differences in hippocampal, PFC, and amygdala volume, and changes in prefrontal–amygdala connectivity [28–30]. These models have also been associated with changes in peripheral stress responses systems, including altered cortisol and autonomic nervous system reactivity to laboratory stressors [31–33], epigenetic changes [34, 35], and increases in markers of inflammatory activity and immune dysregulation [36, 37]. Longitudinal studies tend to provide support for cumulative or general effects [38–40]. A recent longitudinal study from 18 months to mid-adulthood found that cumulative stress rather than physical abuse alone was predictive of adult depressive symptoms [40]. Another study, which followed children from birth to age 37 years, found that childhood stress interacted with current life stress, regardless of type of stressor, to predict diurnal cortisol patterns in adulthood [38]. However, while cumulative models have greatly informed our understanding of the aggregate effects of stress on individuals, they have lacked consistent insight into the neurobiological mechanisms underlying individual differences to children’s responses to stress [14]. This suggests that counting types of stressors alone is not sufficient to explain variation in children’s development outcomes after early life stress.

#### Insights from specific methods

Specific models represent a reaction to cumulative models and an attempt to more precisely identify the neurobiological mechanisms linking early experience to development [20, 41]. These approaches are based in animal models that demonstrate some specificity in the effects of certain types of stressors on neurobiological systems [42, 43]. Based on this evidence, specific models assume that different types of stressors will have distinct and separable effects on developing neural systems. While there are many different variants of this approach [41, 44, 45], an increasingly prevalent one is to conceptualize potential stressors as a lack of expected inputs (i.e., “deprivation”—consisting of things like neglect, food deprivation, and institutionalization) or a presence of direct threat to the child (i.e., “threat”—consisting of things like physical abuse, sexual abuse, and exposure to violence) [46–48].

The rapidly expanding literature taking this approach has provided insight into some of the potential mechanisms supporting the effects of early life stress on development. For example, this literature appears to find more consistent evidence for the association between “threat” and psychopathology being mediated by alterations in stress response systems (including autonomic and HPA reactivity). In contrast, it finds less evidence for the association between “deprivation” and psychopathology being mediated by alterations in stress response systems [49, 50]. However, there are also findings that suggest similar effects of “threat” and “deprivation” experiences on stress response systems and the neural systems supporting them [51–54]. As an example, both threat and deprivation have been linked to negative PFC–amygdala connectivity in late childhood and adolescence [51, 55]. Additionally, both threat experiences such as abuse and deprivation experiences such as neglect have been demonstrated to have specific effects on hippocampal volume [53, 56, 57].

One potential explanation for these commonalities in the effects of different types or categories of stressors is that different types of stressors often co-occur [58, 59]. This co-occurrence creates a number of conceptual issues and makes it difficult to determine if one specific type or dimension of stressor is indeed driving an effect (for extensive discussion see [60]). To illustrate, imagine a study in which a sample of children exposed to physical abuse demonstrate dampened PFC–amygdala connectivity in response to threat. It could be this association is driven by exposure to physical abuse. But, given physical abuse is associated with many other co-occurring risk factors [61–63], it could also be driven by any one of these other risk factors. This makes it difficult to determine what effects are the causal result of just physical abuse, or even if physical abuse itself elicits a neurobiological response. Despite these issues, together general and specific models have provided insight into how early life stress may be shaping neurobiological systems; below, we review commonalities in findings across the two approaches on the development of neurobiological systems.

### Neurobiological consequences of early life stress

While strong arguments have been made for using one type of conceptualization over another [14, 15, 47], careful examination of this literature suggests that there are commonalities in findings across the two approaches. Here, we focus on some general recent themes across this literature with implications for human development. Early life stress is consistently associated with altered functioning of the hypothalamic pituitary adrenal (HPA) axis and autonomic nervous system [33, 54, 64]. These systems are critical to facilitating motived psychological and behavioral responses to the environment, particularly environmental threats and challenges [65, 66]. Additionally, growing evidence suggests that early life stress is associated with alterations in the immune system and inflammatory activity, which is increasingly implicated in producing shifts in individuals’ behavioral responses to their environment [46, 67]. Together, these changes in peripheral physiological systems are critical for facilitating adaptive responses to threat and challenge. In addition, altered activity of these systems is associated with negative mental and physical health consequences after stress exposure [68–70]. The effects of early life stress on these peripheral stress response systems are thought to be a result of altered neural plasticity in circuits integral to stress responses, including the prefrontal cortex (PFC), hippocampus, amygdala, and striatal circuits [15, 71]. There is also a growing corpora of research implicating epigenetic changes in the regulation of many of these effects [34, 72]. Many of these changes have been hypothesized to represent adaptive responses to environments of high threat which become problematic within the broader social context [73, 74]. Below, we review the current state of the literature linking early life stress to altered brain function, and some of the potential hormonal, psychophysiological, neural, and genetic mechanisms thought to support these effects.

#### Neural consequences of early life stress and their proposed mediating mechanisms

##### Alterations in prefrontal–hippocampal–amygdala circuits

Research in both non-human animals and humans suggests that early life stress is linked to pronounced effects on the development of prefrontal–hippocampal–amygdala circuits. These circuits play an important role in facilitating peripheral stress responses through the release of corticotrophin reducing hormone (CRH) and glucocorticoids and regulation of the autonomic nervous system [9, 75]. Additionally, these circuits are implicated in emotion processing, self-regulation, and memory and learning [76–78]. Rodents exposed to abusive maternal behaviors or maternal separation as pups show decreased dendritic arborization throughout the PFC and hippocampus [79, 80]. Experiences of chronic restraint stress in adult rodents result in increased dendritic arborization in the amygdala [81, 82], and there is some evidence for similar effects in the amygdala after experiences of stress as pups [83]. In association with these structural changes, rodents demonstrate modifications in synaptic signaling and epigenetic changes in the hippocampus and amygdala [34, 84–86]. These changes in synaptic structure and signaling are thought to produce increased sensitivity to threat in the environment, through decreased regulation of the amygdala by the PFC and hippocampus [87, 88]. Additionally, they have been associated with increased anxiety and depressive-like behaviors in animals after experiences of early life stress [89–92]. Changes in hippocampal synaptic plasticity have also been linked to altered memory and learning processes, with rodents’ demonstrating reduced spatial memory [93, 94] and enhanced threat learning [95, 96].

The changes throughout the PFC, hippocampus, and amygdala and their associated effects on behavior, memory, and learning appear to be at least partially mediated by chronic exposure to CRH and glucocorticoids induced by chronic stress [93, 97–99]. For example, rat pups exposed to chronic stress in the form of fragmented maternal behaviors demonstrate augmented expression of CRH in the hippocampus and memory deficits. Blocking CRH receptors resulted in improved memory performance and prevented dendritic atrophy in the hippocampus [93]. Chronic maternal separation stress in mice is associated with decreases in glucocorticoid receptor mRNA in the brain, especially so in the amygdala, which is in turn associated with alterations in anxiety-like and social behaviors. Restoring the glucocorticoid receptor mRNA deficit in the amygdala reverses the changes in anxiety and social behavior [100]. Additionally, in male mice, enhanced freezing behavior in the context of a conditioned threat paradigm after exposure to fragmented maternal behaviors can be reversed by blocking glucocorticoid receptors [95].

In humans, similar changes in brain structure and function after experiences of stress in childhood are evidenced in the amygdala, PFC, and hippocampus. Indeed, one of the most reliable findings in children exposed to early life stress is reduced hippocampal volume [29, 53, 56]. Reduced hippocampal volume in children exposed to a range of different types of early life stress, including abuse, neglect, and living in poverty, has been linked to increased symptoms of psychopathology [101–104]. Additionally, changes in hippocampal volume are thought to be associated with deficits in learning processes in children who experience early life stress [7, 105, 106]. A growing literature also indicates that early life stress is associated with changes in amygdala and PFC reactivity to emotional stimuli as well as altered connectivity between the two regions [51, 52, 107]. Cumulative stress, severe neglect from early institutionalization, and abuse have all been associated with heightened amygdala reactivity to emotional images [28, 108, 109]. This heightened reactivity appears to be at least partially a result of altered PFC–amygdala connectivity, leading to increased sensitivity to emotionally salient cues [107, 110, 111]. Indeed, children with a history of maltreatment, which includes emotional, physical, and sexual abuse and emotional and physical neglect, appear to demonstrate atypical connectivity between the amygdala and inferior frontal gyrus [112], and children growing up in poverty is associated with atypical ventrolateral PFC–amygdala connectivity [113]. Longitudinal work suggests that children exposed to various forms of early life stress demonstrate an atypical trajectory of age-related changes in PFC–amygdala connectivity as compared to peers who were not exposed to early life stress [51]. The strength of PFC–amygdala connectivity appears to mediate the relationship between maltreatment exposure and anxiety and depressive symptoms [114, 115]. Structural and functional alterations in PFC–hippocampal–amygdala circuits in individuals exposed to various forms of early life stress suggests that alterations in these circuits play an important role in the relationship between early life stress and its effects on development.

As with non-human animals, there is also evidence that changes in CRH and glucocorticoid function may partially mediate the neural effects described above [34, 54]. Indeed, there is some evidence that humans demonstrate similar epigenetic changes in glucocorticoids to those observed in non-human animals, and these alterations are associated with changes in the hippocampus, symptoms of psychopathology, and altered learning processes [72, 116–118]. Additionally, abnormal hypothalamic pituitary adrenal responsivity is often observed after a variety of experiences of early life stress, including poverty, family violence, maltreatment, and institutional deprivation, although this varies with age [54, 68]. This, in parallel with the animal literature demonstrating that extended exposure to glucocorticoids leads to hippocampal atrophy and dysregulation of the HPA axis [119, 120], has given rise to the hypothesis that chronic activation of the HPA axis through exposure to severe and/or extended stress leads to neural alterations in the PFC, hippocampus, and amygdala. This in turn produces dysregulation in systems responsible for responding to potential threats and challenges in the environment [64, 71]. This dysregulation of stress response systems can lead to increased risk for both mental and physical health issues [121–123].

The effects of early life stress on PFC–hippocampal–amygdala circuitry are thought to be in part related to alterations in emotion processing produced by the types of early inputs children in high stress environments experience. Relative to non-maltreated children, children who experience physical abuse have heightened perceptual and physiological sensitivity to angry facial expressions [124, 125] and are more likely to perceive emotional situations as demonstrating anger as early as preschool age [41]. Physically abused children also more readily categorize faces that are morphed between two different emotions as angry [126] and require less perceptual information to identify faces as angry than non-maltreated children [124]. Additionally, physically abused children show biases to angry faces during cognitive tasks. They respond more quickly to angry faces during a Go/No-Go paradigm [22] and seem to require greater cognitive resources to disengage their attention from angry faces, showing delayed disengagement when angry faces served as invalid cues in a selective attention paradigm [127]. Children who are exposed to extreme threat appear to preferentially attend to and identify facial movements that are associated with threat, such as a scowling facial configuration [125, 128–131], and more reliably track the trajectory of facial muscle activations that signal threat [132]. This close attention to cues of anger likely shapes how abused children understand what facial movements mean. For example, one study found that 5-year-old abused children tended to believe that almost any kind of interpersonal situation could result in an adult becoming angry; by contrast, most non-abused children understand that anger is likely in particular interpersonal circumstances [133].

Studies of maltreated children (including those who experience neglect and other forms of abuse) also show less accurate identification of facial emotions in general [41, 131] and particular difficulty identifying positive emotions [134]. In addition, these children demonstrate abnormalities in the expression and regulation of emotions [135]. Neglected children show delays in perceiving emotions in the ways that adults do [41]. Maltreated children also show higher levels of rumination (repeatedly dwelling on past negative experiences), which has been associated with an attention bias to sad faces [136] and may contribute to risk for depressive symptomatology. The combination of difficulties with emotional recognition, expression, and regulation may increase children’s risk for a broad range of maladaptive outcomes. For example, misreading others’ facial emotion might impair peer interactions, while problematic emotion regulation and expression may contribute to rumination and/or aggressive behavior. The effects of maltreatment on children’s recognition of and attention to emotion are thought to, in part, be shaped by the broader environment in which they are raised. Children who grow up in environments where emotional interactions with caregivers are highly atypical have different developmental trajectories than do those growing in more consistently nurturing environments [8]. Parents from these high-risk families signal emotions unclearly, and express more anger [14, 29, 137, 138]. Together, this suggests that exposure to increased levels of potential threat alters children’s perceptual processes such that they become more likely to perceive situations others may not find threatening as threat, likely resulting in extending activation of prefrontal–hippocampal–amygdala circuits and associated peripheral stress response systems.

##### Alterations in prefrontal–striatal dopaminergic circuits

Recent evidence suggests that early life stress also has a range of negative effects on dopaminergic circuits involved in motivation, specifically those related to reward processing [138, 139]. Animal models of early life stress have been associated with changes in circuits classically implicated in motivation to obtain and pursue rewards, including the ventral striatum, prefrontal cortex, and amygdala [140, 141]. Chronic repeated separation of rodent pups from their mothers alters the number of dopaminergic glial cells, affects rate of cell proliferation and death, and promotes aberrant dopaminergic signaling in the ventral tegmental area and substantia nigra in adulthood [142–144]. Additionally, alterations in maternal care have been associated with reduced connectivity between the PFC and caudate putamen [145] as well as structural and functional alterations in the nucleus accumbens [79, 146]. These changes have been linked to increased anhedonia-like behaviors [147, 148] and altered sensitivity to reward, both hyper- and hyposensitivity depending on the paradigm utilized [149, 150]. As with changes in the hippocampus and amygdala, chronic exposure to glucocorticoids, through interactions with dopaminergic neurons, appears to play an important role in mediating some of these effects [151–153].

In humans, disruptions during reward processing have been observed in the nucleus accumbens, ventral tegmental area, ventral striatum, and PFC after experiences of early life stress [154–157], and these disruptions are associated with depressive and anxiety symptoms in adolescents and adults [158–161] as well as altered reward learning [11, 15]. Specifically, children who experienced maltreatment demonstrate decreased striatal, orbitofrontal cortex, and hippocampal activation during reward learning [157], and children with high early life stress demonstrate decreased activation of the putamen and insula when anticipating future losses [138]. Additionally, in children exposed to early life stress, ventral striatal activation has been demonstrated to mediate variation in reward related learning [162]. Importantly, these circuits are highly connected with both the amygdala and prefrontal cortex, which together play a key role in psychological and behavioral responses to stress, emotional and social learning, and self-regulatory processes [163, 164]. These disruptions likely then place children at increased risk for maladaptive behaviors, along with negative mental and physical health outcomes later in life.

#### Summary

Despite the relationships between early life stress and alterations in both PFC–hippocampal–amygdala and dopaminergic reward circuitry outlined above, we still understand relatively little about how these changes are associated with altered learning and behavioral patterns and how they increase risk for mental and physical health disorders and disease. Additionally, it is still unclear which changes are important for different types of health risks and what supports individual differences in children’s outcomes after experiences of early life stress. While the frameworks for conceptualizing early life stress outlined above were developed to try and address this question, there are still many findings that are not fully accounted for, suggesting that additional factors may also be critical for shaping children’s neurobiological responses to stress.

### Promising future approaches to elucidating the mechanisms of early life stress

A commonality across both general and specific models is a focus on identifying types of events a child may or may not be exposed to that meet the criteria of a stressor based on some outside determination (be it criteria set by child protective services for abuse or neglect, economic guidelines for poverty, or researchers determination that one thing represents a stressor over another). But an additional insight into the neurobiological mechanisms underlying the effects of early life stress may lie with an individual child’s interpretation or perception of those events. Even in non-human animal models, which do evidence specificity in responses to stress [165, 166], there are a range of individual differences in behavioral responses to the same type of stressor [167]. These individual differences in behavior are supported by different physiological and neural mechanisms [168–170]. Similar variability in response to adverse events is observed in humans across neurobiological stress responses systems [66, 171–173], and this variability has been linked to differential health behaviors and symptoms [174–176].

This range of variability in neurobiological responses to similar types of stressors has led to the proposition that it is not the type or features of an adverse event, but rather the organisms’ perception and interpretation of that event, that that has different effects on neurobiological systems [166, 177, 178]. There is now a wealth of research in adults demonstrating that individual variability in neurobiological responses to stress is informed through the assessment of factors that shape perceptions and interpretations of stress [10, 179, 180]. For example, individual variability in cortisol responses to social speech stress is positively related to how individuals rate their perceived stress during the stressor [175]. Shifts in how humans and animals perceive the controllability and predictability of a stressor will change their physiological responses to that stressor [181–184]. In humans, individual differences in perceptions of control have been linked to differential cortisol responses to acute laboratory stress, differences in brain volume, and differences in brain reactivity to stress in regions including the hippocampus, amygdala, and prefrontal cortex [185–187]. Additionally, perceived adversity, and its associated neurobiological responses, can occur in the absence of any specific identifiable environment event through rumination over previous experience or events or anxiety about future events [188–190]. Recent evidence in children suggests a similarly important role for perception in variability in stress responses. One study utilizing machine learning approaches found that event exposures are not highly predictive of children’s outcomes [191] and another found reported exposure to abuse or neglect is more predictive of children’s mental health outcomes than exposure identified through court reports [192].

There is a growing literature suggesting that the chronicity, developmental timing, and intensity of adversity exposure are important factors shaping the effects of adversity on children [68]. In animal research, the precise timing of when during development a stressor occurs can be tightly controlled, and has demonstrated strong effects as described in a number of recent reviews [46, 68, 193, 194]. However, the developmental period in which adversity occurs is tightly intertwined with the chronicity of adversity (that is, adversity that begins early in a child’s life may be longer lasting and chronic than adversity that begins later in a child’s life), which also demonstrates profound effects on variability in responses to stress [82, 195]. Children with high scores on the Life Stress Interview (LSI), which quantifies the intensity of children’s stress exposure, have smaller amygdala and hippocampal volumes than children exposed to less intense levels of early life stress [29]. Children with high levels of early life stress demonstrate altered activation in circuits involved in value processing during anticipation of rewards and losses [138]. Retrospectively reported severity of early stress exposure in childhood has also been associated with increased dorsal medial PFC responses to a social stressor [196] and altered global connectivity of the ventrolateral PFC [197]. Both severity and amount of maltreatment in children have been linked to epigenetic changes of the glucocorticoid receptor gene [198]. Additionally, variations in intensity of early adversity appears to modulate HPA responses with retrospectively reported intensity of stress, rather than type of stress, during early childhood being associated with increased levels of cerebrospinal fluid (CSF) CRH [199], and increased cortisol responses to acute social speech stress [200]. Children’s rated intensity of adversity also interacts with age to predict cortisol awakening responses [201].

Another potential factor in shaping child development may be features of the early environment such as predictability and contingent responding of caregivers (or, alternatively, chaos and lack of stability) [140, 202]. Parent–child relationships are stereotypically repetitive, highly predictable, and marked by contingent parental responses. In normative contexts, adult caregivers reliably respond to infant cries, comfort a child who is hurt, and provide support to a child who is dysregulated [203, 204]. Lack of predictable and contingent input from caregivers affects children’s expectations of the environment, leading to uncertainty and perceptions of vulnerability [11, 137]. While there is limited research directly assessing variation in the predictability of children’s environments, there is a growing literature that suggests it has the ability to provide great insight into the mechanisms underlying experiences of early life stress. Longitudinal research assessing early influences on adolescents’ externalizing behaviors finds that unpredictability of the environment during childhood, quantified using changes in maternal employment, changes in residence, and changes in cohabitation, is associated with increased externalizing behaviors in adolescence while SES was not related [205]. Recent research in rodents suggests that these observed effects are a result of altered functioning in prefrontal–hippocampal–amygdala circuits, finding that unpredictable maternal inputs are associated with altered connectivity between the medial prefrontal cortex (PFC) and amygdala [91] as well as decreased dendritic arborization in the hippocampus [206] beyond effects produced by types of maternal inputs. These effects are linked to PTSD and depressive-like behaviors as well as deficits in learning [140]. Together, this body of work suggests that variation in the predictability, stability, and/or degree of contingent responding of adult caregivers to the needs of the developing child is a factor in shaping children’s responses to adversity through alterations in prefrontal cortical and subcortical stress response circuits. It indicates that assessment of predictability of early environments, along with exposure to negative events, has the potential to provide increased insight into individual differences in the neurobiological effects of early adversity on child development that is not captured when focusing solely on types of adverse events.

Last, increasingly research supports a role for perceived safety in contributing to variations in children’s responses to stress. Safety/security in early childhood has been characterized in a variety of different ways, with things such as parental presence/adult “buffering,” sensitivity, responsivity, and support thought to be cues of safety, and lack of parental input, through isolation, maternal separation, or neglect, or abusive parenting behaviors being cues of lack of safety [207–209]. Cues of safety early in development play an important role in engaging the prefrontal circuits that inhibit threat response circuits, which will have implications for how children perceive and interact with their environment later in life [210]. Indeed, evidence from non-human primate and rodent models supports this finding that early parental presence plays an important role in inhibiting neurobiological threat response systems, with both rodent pups and infant primates demonstrating reduced glucocorticoid release and decreased amygdala activation in the presence of the mother [207, 211]. However, in cases of abusive maternal rearing, maternal presence does not appear to exhibit buffering effects. Under these circumstances, rodent pups and primate infants demonstrate enhanced glucocorticoid responses to stress [207, 212] as well as alterations in both the structure and function of the amygdala and prefrontal cortex [213–215]. From this literature, it is clear that parental presence, a salient early cue of safety, is important to supporting typical development of the neurobiological stress response systems.

There is some evidence indicative of similar early regulatory effects of parental presence on the development of stress response systems in humans [208, 216]. In parallel to the rodent and primate literatures, parental presence has been demonstrated to dampen both cortisol [217, 218] and amygdala reactivity [219] to stress in children. Presentation of parent voice during speech stress has been associated with faster cortisol recovery post-stressor [218], suggesting that parent support does not necessarily need to be physical to buffer children’s responses to stress. There is also evidence that early adversity is associated with altered prefrontal–amygdala connectivity, and these alterations have been linked to children’s risk for psychopathology [51, 114, 220]. This points to disruptions in the development of these circuits in children lacking early cues of safety that have implications for their behaviors and mental health. However, in cases of adversity where children still receive high levels of support from their parents, these effects are mitigated, with adolescents living in poverty showing altered connectivity in prefrontal cortical networks involved in executive functioning and emotion regulation, but not if they reported having high levels of parent support [221]. Additionally, support provided by other adults or peers may diminish some of the bio-behavioral effects of adversity, with reported social support from family and friends being associated with reduced risk of psychopathology in children who experience maltreatment [222, 223]. This suggests that, at least in humans, individuals outside of the parent–child relationship may be able to supplement these safety cues when they break down. Consistently incorporating assessment of factors that represent early cues of safety, such as parental support, when studying how children respond to early adversity, has the potential to greatly illuminate the neurobiological mechanisms through which negative environments shape development.

#### Summary

There is consistent evidence that early life stress exposure changes neural plasticity and function, and these changes have implications for children’s mental and physical health across the lifespan. Studies assessing differential effects of events along with timing and intensity of events, predictability and contingency of environmental inputs, and perceptions of safety and social support suggest that these factors differentially shape biological systems involved in stress. Of course, it is the case that there are probably bidirectional effects between exposure to potentially stressful events shaping children’s perceptions of their environment in turn resulting in children perceiving their environment as more stressful. For this reason, it may seem like it is easier to establish causality through approaches focusing on identifying events and their associated outcomes. However, while events themselves likely contribute to how children perceive their environment, approaches which focus only on events are missing a multitude of other sources of variation in these perceptions. Further incorporation of factors that may shift how individuals interpret their environment, in combination with event based methods of assessment of stress and rigorous longitudinal studies with assessments at multiple timepoints, has the potential to provide increased insight into the specific neurobiological mechanisms influencing children’s development. This type of approach can aid in identifying what may produce resiliency to negative mental and physical health outcomes in children who experience early life stress.

## Conclusions

In this article, we have highlighted recent research speaking to the neural mechanisms underlying the effects of early life stress on development. The existing literature supports effects of early life on the development of the prefrontal cortex, hippocampus, hypothalamus, and amygdala, along with communication across those areas, in ways that produces increased vulnerability to mental and physical health disorders later in life. These changes appear to be at least partially mediated through hormonal and neuropeptide alterations in the HPA axis along with interactions with genetic and epigenetic factors. Additionally, there is increasing evidence for a role of dopaminergic reward circuits in these relationships. However, to date, we still lack a good understanding about how these changes come about, what aspects of the child’s environment produces these changes, and, given not all children who experience early life stress develop later psychopathology, what their role is in individual differences in children’s outcomes after early life stress.

## Data Availability

Not applicable.

## Abbreviations

PFC: Prefrontal cortex
HPA: Hypothalamic–pituitary–adrenal
CRH: Corticotropin-reducing hormone
CSF: Cerebrospinal fluid
